# Book Review: The Educator's Guide to LGBT+ Inclusion: A Practical Resource for K-12 Teachers, Administrators, and School Support Staff

**DOI:** 10.3389/fpsyg.2021.692343

**Published:** 2021-07-15

**Authors:** Alex Siu Wing Chan

**Affiliations:** Department of Applied Social Sciences, The Hong Kong Polytechnic University, Hong Kong, China

**Keywords:** social inclusion and exclusion, LGBT+, school support intervention, educational system, guidance

Harassment, absenteeism caused by the absence of protection in schools, and ensuing suicidal thoughts are all dramatically higher among lesbian, gay, bisexual, and transgender (or sometimes questioning) (LGBT+) adolescents than among non-LGBT+ adolescents. Consequently, numerous K-12 students in the United States are struggling unnecessarily, while numerous educators have yet to figure out how to deal with the problem. LGBT students experience bullying, such as violent acts, alienation, ridicule, or even death threats, leading to a sense of insecurity, skipped learning time, and lower likelihood of educational excellence (Santos et al., 2020). LGBT adolescents are more likely than anyone to attempt suicide as a result of such harassment in school environments. The issue is addressed in this text (Tilley et al., 2020). This relatable and simple book directs instructors, educators, leaders, and school personnel toward effective and validated approaches to establish positive academic settings, adjust educational policies, improve syllabuses, and more successfully serve LGBT+ adolescents while they study, by spelling out guiding principles and providing expert advice for fostering LGBT+ inclusive learning in classrooms.

This book, which includes actual experiences and examples, a checklist, and additional materials, allows experts across a wide range of educational disciplines to incorporate basic principles into their daily encounters with learners, parents, and colleagues in order to foster a general learning atmosphere that cultivates a supportive, inclusive, and accepting community for everyone. This book might be seen by observers, administrative groups, as well as the whole school districts as part of their lessons. Shane offers a powerful framework for K-12 teachers in this text. Her book is a helpful resource for students and staff looking to increase cultural awareness and incorporate guiding principles that benefit LGBT+ learners, households, and employees.

Shane teaches people how to avoid discriminating against LGBT+ individuals in various contexts. She specifies expressions, examines the significance of acceptable and offensive words, and discusses how to talk compassionately to and about LGBT+ individuals. Shane gives a straightforward, concise description of social status and power dynamics in an objective way that piques people's interest in further examining the issue. Hence, Shane offers examples involving people and their circumstances. Such events are based on actual events which occur in school districts and frequently frustrate inexperienced teachers. Shane propose a new strategy, encouraging people to view the events from the point of view of individuals, decision-makers and district authorities, and from the angle of the established practices and guidebooks used by school districts. She subsequently offers a “Guidance” chapter about every case, which includes LGBT+ good practices criteria.

The final portion of this text attempts to put information into practice. It offers teachers advice and guidance for recognizing their capabilities and creating a strategy to tackle locations in which their culture has been developing. Shane also provides template emails to communicate with a principal or district leader in order to initiate a dialogue about transition. She offers a comprehensive appendix with a wealth of tools for teachers, such as texts for learners across all levels of education. Such tools and tactics lay the groundwork for campaigning.

Shane's book is a great resource for students and staff. School counselors, student volunteers, and human services mentors who are training new groups of social workers can obtain valuable material to meet the demands of LGBT+ learners, households, and schools. Shane's book, which was influenced by her own doctoral experiences in education and public assistance, serves as a basis for counselors to interact with academic contexts. This information could be adopted by social workers to educate educators, supervisors, and school officials. The material may be utilized in organized lectures or reading groups to progress on the possible circumstances and develop improvement strategies. With case studies, the events offer a deeper level of understanding of micro-level approaches. Within those obstacles, many school counselors would identify themselves as well as respective school districts. More importantly, Shane offers a chance for campaigning and analysis on mezzo-macro approaches which could alter school culture.

Years back, the LGBT+ group was marginalized, but now, the LGBT+ population is demanding equal rights. Religion and tradition have a significant impact on all individuals, and their viewpoint on LGBT+ is accepted by the general public. The LGBT+ culture is one-of-a-kind, and often people fail to realize that these individuals are far more valuable than they historically assumed. Several approaches are not directly applicable to LGBT+ individuals (Chan, 2021). For example, in an educational setting, LGBT+ children sometimes encounter verbal or physical abuse and humiliation. LGBT+ learners are more vulnerable to unfavorable academic consequences when faced with substantial challenges such as bullying, violence, and the absence of good examples to follow. Nonetheless, LGBT+ learners have to have allies. Inclusion, dignity, affection, and thoughtfulness must be upheld in educational institutions. This text, I presume, will assist policymakers in developing a more accepting school atmosphere for LGBT+ learners. Building a positive atmosphere for LGBT+ learners enhances learning results among all students besides those who are classified as LGBT+. This text may put a spotlight on the despair of the marginalized LGBT+ community and the unreasonable rejection of them within the academic context. Therefore, ongoing development in the field is obviously required to guarantee that every single student is seen as having the right to an education that is pleasant, inclusive, and devoid of discrimination, and that LGBTQ-inclusive education is an integral component in building those environments (Chan, 2021).

Eventually, Shane's book, The Educator's Guide to LGBT+ Inclusion: A Practical Resource for K-12 Teachers, Administrators, and School Support Staff, provides a chance for counselors to assist school environments *via* a thoughtful framework of rationality and practice for building more positive academic cultures for LGBT+ individuals. To achieve a meaningful and comfortable life, it is important to maintain self positive mental health (Chan et al., 2021). This book ought to be read by all educators and school leaders, and it must be part of the curriculum in undergraduate education and community services training. This text is particularly significant as it discusses the roots and effects of LGBTQ discrimination. I highly recommend it since it advocates the concept of social inclusion in the educational system.

## Author Contributions

The author confirms being the sole contributor of this work and has approved it for publication.

## Conflict of Interest

The author declares that the research was conducted in the absence of any commercial or financial relationships that could be construed as a potential conflict of interest.
